# Enhancing knee osteoarthritis diagnosis with DMS: a novel dense multi-scale convolutional neural network approach

**DOI:** 10.1186/s13018-024-05352-0

**Published:** 2024-12-19

**Authors:** Di Zhang, Yuting Dong, Yao Xu, Junhui Qian, Miaoyu Ye, Qiang Yuan, Jian Luo

**Affiliations:** 1 School of Acupuncture and Tuina, Chengdu University of Traditional Chinese Medicine, Chengdu, China; 2 Department of Tuina, Hospital of Chengdu of Traditional Chinese Medicine, Chengdu, China

## Abstract

**Background:**

Osteoarthritis (OA) of the knee is a prevalent chronic degenerative joint condition that is having a growing impact on a global scale., posing a challenge in diagnosis which is often reliant on time-consuming and error-prone visual analysis by physicians. There is a critical need for an automated, efficient, and accurate diagnostic method to improve early detection and treatment.

**Methods:**

We developed a novel Convolutional Neural Network (CNN) module, Dense Multi-Scale (DMS), an advancement over Multi-Scale Convolution (MSC). This module utilizes dense connections in convolutions of varying sizes (1 × 1, 3 × 3, 5 × 5) and across layers, enhancing feature reuse and complexity recognition, thereby improving recognition capabilities. Dense connections also facilitate deeper network architecture and mitigate gradient vanishing problems. We compared our model with a standard baseline model and validated it using an unseen-data test set.

**Results:**

The DMS model exhibited exceptional performance in unseen-data tests, achieving 73.00% average accuracy (ACC) and 92.73% area under the curve (AUC), surpassing the baseline model’s (DenseNet) 63.52% ACC and 88.76% AUC. This highlights the DMS model’s superior predictive capability for knee OA.

**Conclusion:**

The DMS model presents a significant advancement in predicting and grading knee OA, holding substantial clinical importance. It promises to aid radiologists in accurate diagnosis and grading, and in choosing appropriate treatments, thereby reducing misdiagnosis and patient burden.

## Introduction

Osteoarthritis (OA), a highly prevalent joint disorder on a global scale, has become a significant public health concern [1], affecting over 240 million people globally [2] and incurring an annual treatment cost of up to 303 billion dollars [3]. The pathogenesis of knee OA involves a disruption in the balance between joint tissue breakdown and repair, leading to loss of cartilage, subchondral bone remodeling, osteophyte formation, and damage to surrounding muscles, which manifest as joint pain, stiffness, and functional impairment [4]. Various factors, such as aging, obesity, joint injuries, and insufficient bone density, are recognized as risk factors for OA [5]. Research findings suggest that osteoarthritis of the hip and knee are significant contributors to worldwide disability [6]. In China, the prevalence of OA symptoms is relatively high among people aged 60 and above, averaging 19.4%, with 10.3% in women and 5.7% in men [7]. As the worldwide population continues to age, the prevalence of osteoarthritis is on the rise, leading to a notable impact on patients’ quality of life and presenting risks to their mental well-being. Research has shown that the relative risk of depression and anxiety in OA patients has increased by 1.1 and 1.35 times, respectively [8]. Therefore, implementing effective measures to enhance the treatment and prevention of OA, improving patients’ quality of life, and alleviating societal burdens are critical to advancing global public health endeavors.

Currently, the diagnosis of osteoarthritis heavily relies on imaging studies, an indispensable and crucial component of the diagnostic process. Radiology plays a vital role in the diagnosis, therapeutic management, and scientific research of osteoarthritis [9]. Among the various modalities, MRI, CT, and X-ray are the most common, with X-ray being considered the “gold standard” for evaluating joint structures [10]. In clinical practice, physicians commonly utilize the Kellgren and Lawrence (KL) grading system to evaluate the severity of knee osteoarthritis, stratifying the advancement of the condition into grades ranging from 0 to 4 [11]. This assessment necessitates a meticulous examination of the patient’s knee X-ray images to identify signs of pathology such as joint space narrowing, cartilage damage, and osteophyte formation [12]. These signs are crucial in determining the progression of knee osteoarthritis and assist in devising personalized treatment plans. However, the manual evaluation of X-rays has its limitations. This process generally requires experienced physicians to perform manual analyses, demanding a high level of expertise and experience. Insufficient expertise may lead to misdiagnoses or missed diagnoses, adversely affecting patient treatment and recovery. Additionally, this manual analysis is time-consuming and labor-intensive. The entire process, from image acquisition to physician analysis, is lengthy, affecting the efficiency of patient care. In high-volume medical environments, such inefficiencies may result in delays in the diagnosis and treatment of patients. To overcome these challenges, modern medicine is dedicated to developing more advanced, efficient imaging technologies and fully automated, effective assistive grading methods. These new techniques and methods aim to enhance the accuracy and efficiency of diagnoses, lighten the workload of physicians, and better meet patient needs, thereby advancing the field of osteoarthritis diagnostics.

Since the rapid advancement of artificial intelligence and deep learning technologies, deep learning has become increasingly useful in the diagnosis of arthritis. In recent years, researchers have developed various innovative machine learning algorithms, paving new pathways for the accurate diagnosis and quantitative assessment of osteoarthritis. For instance, Abdelbasset Brahim et al. [13] introduced a polynomial logistic regression (MLR) model for analyzing and classifying knee joint X-ray images, facilitating the quantitative assessment of knee osteoarthritis (KOA). Norman et al. [14]suggested the implementation of a completely automated algorithm. that employs template pattern matching techniques, combined with manually cropped images and fully connected layers to transform demographic data into vectors for predicting the severity of OA. Thomas et al. [15] created a comprehensive interpretable mode that inputs complete X-ray images and predicts KL scores with cutting-edge precision. Lau et al. [16]developed a machine learning model based on X-ray images obtained from TKA patients, with the help of ImageNet and Xception models. Additionally, they integrated clinical parameters of TKA patients, creating another system that uses random forest classifiers for osteoarthritis classification based on clinical information.

**Fig. 1:**
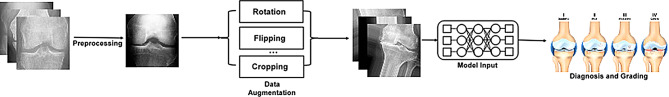
The workflow of this study

This study presents the introduction of a deep learning model known as Dense Multi-Scale (DMS), which serves as an improvement upon the Multi-Scale Convolution (MSC) model. We observed significant performance improvements when using MSC-integrated CNN models for the prediction of osteoarthritis. However, these models still faced challenges in effectively capturing certain detailed features and preventing gradient vanishing when increasing network depth. To address these limitations, we integrated DenseNet’s dense connectivity strategy within the MSC convolutions and across different convolutional layers. This not only enhanced the recognition of detailed features but also effectively resolved the gradient vanishing issues associated with deeper network structures. The DMS model proposed in this study demonstrated exceptional performance in unseen-data testing and comparison with traditional models. Figure 1 outlines the workflow for this study.

## Methods

### Database

**Fig. 2:**
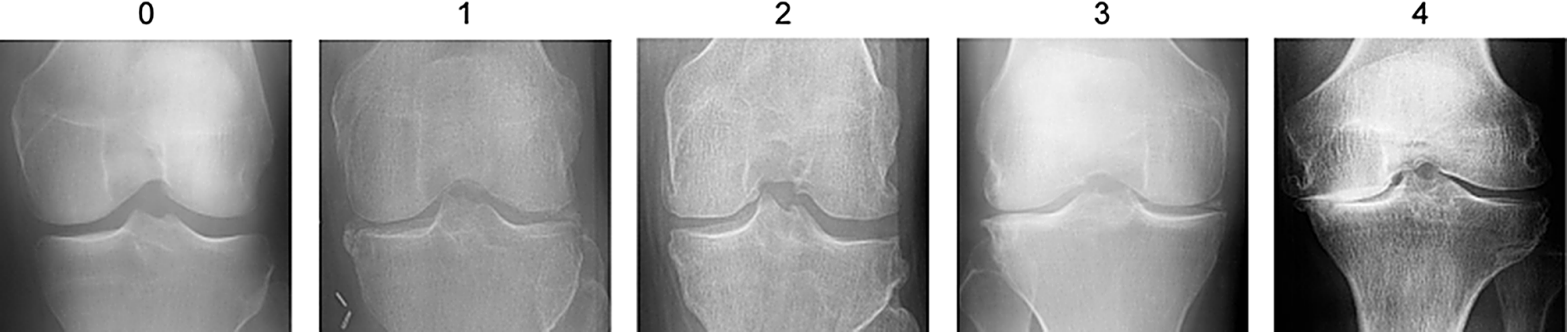
Display of each classification in the OAI database

The data used in this study were obtained from the Osteoarthritis Initiative (OAI) database, designed to investigate knee health and risk factors for knee OA [17]. The OAI enrolled 4,769 males and females aged between 45 and 79 years at the onset of the 4-year study period. Data were collected at four urban clinical sites. Individuals with rheumatoid arthritis (RA) or inflammatory arthritis were excluded, as were those who had undergone bilateral knee replacement. The OAI study complied with the Health Insurance Portability and Accountability Act (HIPAA) and institutional review board (IRB) regulations, with all participants providing informed consent. The sample was divided into three subcohorts: a control group (with no knee OA and no risk factors for knee OA; *n* = 122), an incidence group (with no symptoms of knee OA but with risk factors; *n* = 3284), and a progression group (with symptomatic knee OA; *n* = 1390). In this study, focusing on unimodal recognition, we selected a subset of X-ray images (totaling 9,786, with 5,778 for the training set) and categorized them into five grades. Grade 0 represents normal, while grades 1 to 4 indicate the presence of knee OA, with increasing severity as the grade number increases. The pictures of different classifications in the OAI database is shown in Fig. 2.

### Data preprocessing

In the preprocessing stage, to mitigate the impact of noise on model performance, we first employed a Gaussian filter for noise reduction across all images. Subsequently, the Sobel operator was applied for edge detection, and an accumulation process was utilized to enhance the clarity of image edges and detail information. Furthermore, we implemented histogram equalization to augment the contrast, thereby making the bone structures and soft tissues more distinct and discernible in the images.

Given the data imbalance observed in our chosen dataset (for instance, the training set contained 2,286 images in class 0, while class 4 had only 173 images), this study employed data augmentation techniques to enhance the model’s generalization capability and its ability to handle atypical inputs, as well as to balance the dataset. Specifically, we utilized various data augmentation methods, including rotation, cropping, and flipping, to achieve a more balanced distribution of images across different categories. The combined application of image preprocessing and data augmentation effectively enhanced the model’s performance, improved the accuracy of predictions, and significantly mitigated the adverse impact of data imbalance on the model’s predictive capabilities.

### Model construction

#### Dense multi-scale (DMS) model

The concept of Multi-Scale Convolution (MSC) was introduced within the context of deep Convolutional Neural Networks (ConvNets), first proposed by Liao et al. in 2015 [18]. This approach employs convolutions and filters of multiple scales within the same convolutional layer, fostering competition among a group of multi-scale convolutional filters. Inspired by the inception module, MSC aims to enhance deep ConvNets by preventing filter co-adaptation and encouraging the formation of multiple sub-networks within the same model, which assists in training complex learning problems and reducing the dimensionality of multi-scale filter outputs. Dense Connection, a concept introduced by Huang et al. in 2016 [19], is used to construct Dense Convolutional Networks (DenseNet). In this architecture, each layer is directly connected to all preceding layers, meaning that every layer receives feature maps from all previous layers as input. This structure significantly increases inter-layer information flow, reduces the number of parameters, and enhances the network’s efficiency in learning features. However, the processing of multi-scale features by MSC can significantly increase model depth, leading to issues such as gradient vanishing.

**Fig. 3:**
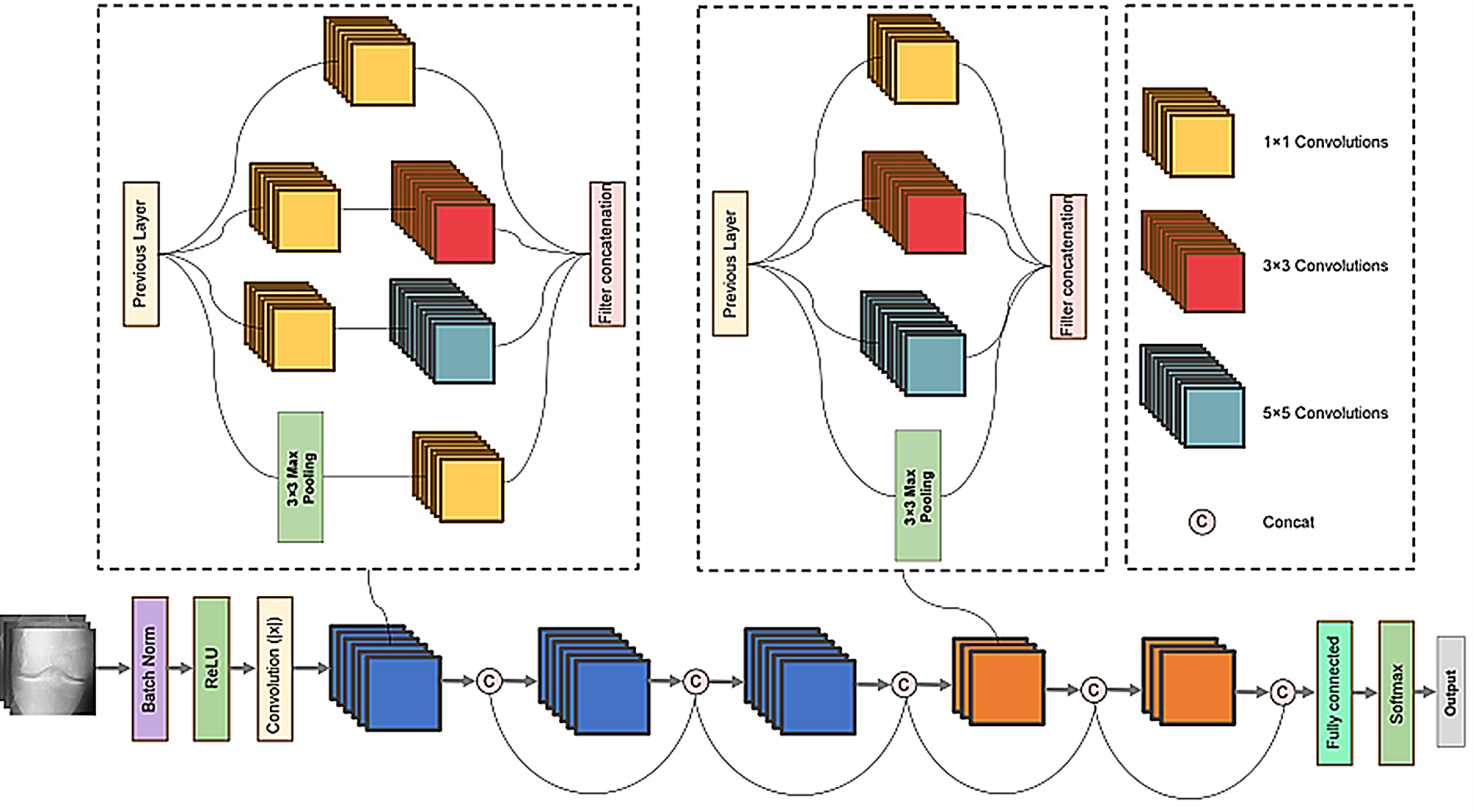
Construction of Dense Multi-Scale model

Given the limitations of MSC and the effectiveness of DenseNet in alleviating gradient vanishing and enhancing detail feature extraction, this study introduces a novel approach that integrates these two technologies. We incorporated DenseNet’s dense connectivity strategy into the convolutions of sizes 1 × 1, 3 × 3, 5 × 5, and across different convolutional layers, resulting in a new architecture named Dense Multi-Scale (DMS). DMS leverages dense connections to augment feature reuse, enabling the model to capture more complex and fine-grained features, thereby significantly enhancing its recognition capability. Moreover, dense connections between layers aid in constructing a deeper network structure, effectively preventing the issue of gradient vanishing. The architectural diagram of the DMS model is illustrated in Fig. 3.

Overall, the advantages of DMS are manifested in the following aspects:

- Enhanced Feature Reuse: The core concept of dense connectivity is to ensure that features from all preceding layers are accessible to subsequent layers, meaning that each convolution in the DMS structure can utilize feature maps from all prior layers, significantly enhancing feature reuse.
- Deeper Networks with Parameter Efficiency: Due to feature reuse, DMS allows the design of deeper network structures without a substantial increase in parameters.
- Mitigating Gradient Vanishing: Dense connections ensure direct gradient flow to each layer, effectively alleviating the common problem of gradient vanishing in deep networks.
- Enhanced Feature Propagation: Features can propagate directly from earlier to later layers, enabling the network to capture richer information.
- Strengthened Feature Learning Capability: Combining the multi-scale attributes of MSC and the dense connections of DenseNet, DMS effectively learns multi-level features.
- Reduced Overfitting Risk: The parameter efficiency of dense connections leads to relatively fewer parameters in the model, lowering the risk of overfitting.
- Accelerated Training Process: Direct feature transmission and easier gradient backflow accelerate the training process.

Through comparative experiments and validation, the DMS model demonstrated superior efficacy compared to traditional models. It more accurately focuses on critical areas within the images and extracts a richer set of detail features, thereby significantly enhancing the model’s ability to recognize and predict knee OA.

#### Baseline models

To enhance the objectivity in assessing the performance of DMS in predicting and grading knee OA, this study constructs a comparative framework using established baseline models including Inception, VGG, EfficientNet, DenseNet, and MobileNet. This comparison is designed to demonstrate the superiority of DMS relative to these widely utilized models in the research community.

The Inception model, initially proposed by Szegedy et al. in 2014 [20], is based on the concept of employing convolutional kernels of various sizes within the same layer, enabling the capture of multi-scale features in images. This design effectively broadens and deepens the network while maintaining computational efficiency. The architectural innovation of the Inception model has led to significant success in image recognition and classification tasks and has been extensively applied in medical image recognition [21–23].

The VGG model, developed by Simonyan and others from the University of Oxford in 2014 [24], enhances performance through increased network depth (up to 19 layers) and the stacking of 3 × 3 small convolutional kernels. Despite its simplicity, this structure is highly effective, particularly in large-scale image recognition tasks. The VGG network has played a pivotal role in understanding the characteristics of deep convolutional networks, influencing subsequent architectural designs in deep learning and its application in the medical field [25, 26].

EfficientNet, introduced by Tan et al. of the Google Brain team in 2019 [27], brought a key innovation with the introduction of a systematic model scaling method known as compound scaling. By scaling the network’s depth, width, and input image resolution simultaneously and proportionately, EfficientNet achieves significant performance improvements with high efficiency. This approach has led EfficientNet to achieve leading performance in various standard image recognition tasks, maintaining a relatively small model size and computational complexity. This model has also found widespread application in the medical domain [28, 29].

MobileNet, designed for mobile and embedded devices, is a lightweight deep learning convolutional neural network architecture introduced by Howard and colleagues at Google [30]. Its defining feature is the use of depthwise separable convolutions, significantly reducing the model’s parameter count and computational complexity while maintaining robust performance. This makes MobileNet particularly suited for environments with limited computational resources. Its lightweight design, which allows for efficient and rapid inference, is also crucial in medical research, thus making it a broadly applied model in the medical domain [31, 32].

By benchmarking against these extensively applied and validated models, the reliability and excellence of our proposed model can be affirmed. Further experimentation is necessary to substantiate the superior capabilities of our model in the field.

### Model evaluation

**Fig. 4:**
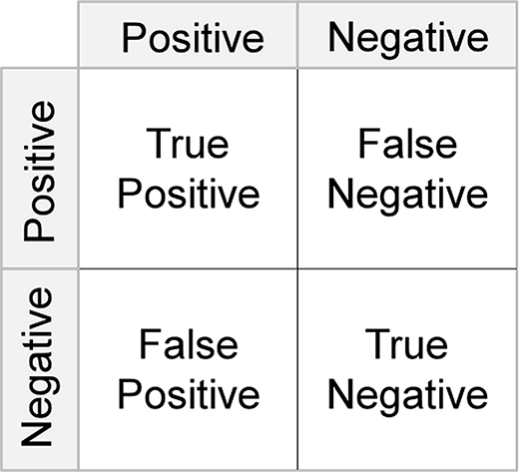
The confusion matrix

For a thorough and unbiased evaluation of the DMS model introduced in this study, the analysis utilizes a set of standardized metrics: Accuracy (ACC), Precision (PRE), Recall (REC), and the Receiver Operating Characteristic (ROC) curve with its corresponding Area Under the Curve (AUC). These metrics are computed from the values of True Positives (TP), True Negatives (TN), False Positives (FP), and False Negatives (FN), which is called confusion matrix, and the meaning of confusion matrix is shown in Fig. 4.

Accuracy (ACC): This metric reflects the overall correctness of the model by measuring the ratio of correctly identified instances (both true positives and true negatives) to the total number of instances. The formula for ACC is:

1 $$\:ACC=\frac{TP+TN}{TP+FP+TN+FN}$$

Precision (PRE): Precision assesses the model’s ability to correctly predict positive instances out of all instances classified as positive. This metric is particularly critical in areas where the cost of a false positive is high, such as in medical diagnostics. PRE is determined by the following formula:

2 $$\:PRE=\frac{TP}{TP+FP}$$

Recall (REC): Also referred to as Sensitivity, recall measures the model’s capability to correctly identify actual positive cases. It is a crucial metric in situations where missing a positive instance could have severe implications. The REC formula is:

3 $$\:REC=\frac{TP}{TP+FN}$$

The Receiver Operating Characteristic (ROC) curve is an essential tool for visualizing the trade-off between the True Positive Rate (TPR) and False Positive Rate (FPR) at various threshold settings. A model with high sensitivity and specificity will have an ROC curve near the top left corner of the plot. The Area Under the Curve (AUC) provides a single value summarizing the model’s ability to distinguish between positive and negative classes across all possible thresholds. A model with perfect classification ability will have an AUC close to 1.

## Results

### Experiment set up

In this paper, we conducted a series of extensive experiments for parameter optimization. In the model parameter settings, we applied the gradient threshold method for tuning parameters. The results of these experiments revealed that setting the learning rate at 0.0001 and the batch size at 64 enables the DMS model to reach convergence with the best outcomes after 50 training epochs, while avoiding overfitting. Additionally, for the sake of objectivity, the parameter tuning approach for the baseline models was aligned with that of the DMS model. All models were selected to undergo an appropriate number of training epochs to achieve full convergence without any evidence of overfitting.

The experimental framework for this study was meticulously established within a computing environment running Windows 11 Professional Edition. Computational tasks were carried out using Python 3.7.0. The research extensively utilized Tensorflow-gpu 2.6.0 for the development of deep learning models. Additionally, Scikit-learn and Sklearn 0.0.post1 were integral for implementing machine learning algorithms and managing data processing tasks, ensuring robust and accurate model training. Scientific computations were handled using scipy 1.10.0. For data visualization and the presentation of results, matplotlib, a widely-used library for creating static, interactive, and animated visualizations in Python, was employed. In terms of hardware, the experiments capitalized on the capabilities of an Intel Core i5 12,400 F CPU. This processor, with a base frequency of 2.5 GHz and a maximum turbo frequency of 4.40 GHz, comprises six cores and twelve threads, providing a balance of efficiency and power for computational tasks. Complementing the CPU, an NVIDIA GeForce GTX 3060 GPU, equipped with a 12GB memory capacity and a 192-bit memory bus width, was utilized.

## Result of unseen-data validation

In this study, to validate the proposed DMS model’s capability in handling unseen data, we divided 20% of data as test setting to conduct unseen-data testing. The model achieved full convergence without indications of overfitting after 50 training epochs, as shown in S5 Fig (a). Moreover, the DMS model exhibited superior performance, with an average ACC of 73.00% and AUC of 92.73%. Remarkably, it achieved prediction accuracies of 91.89% for grade 3 and 97.15% for grade 4 classifications, outperforming the baseline models, the ROC of DMS is shown in S5 Fig(b). This demonstrates significant implications for the prediction and grading of knee OA. The model’s various evaluative metrics are presented in Table 1.

**Table 1:** The performance of different models

	Mean ACC	Mean PRE	Mean REC	Mean F1
New	73.00%	70.40%	75.70%	75.87%
Inception	62.37%	62.62%	64.47%	61.70%
VGG	49.34%	51.25%	47.54%	43.62%
EfficientNet	46.80%	44.73%	46.16%	42.37%
Dense	63.52%	66.64%	64.82%	64.60%
Mobile	49.57%	67.61%	52.07%	53.56%

**Fig. 5:**
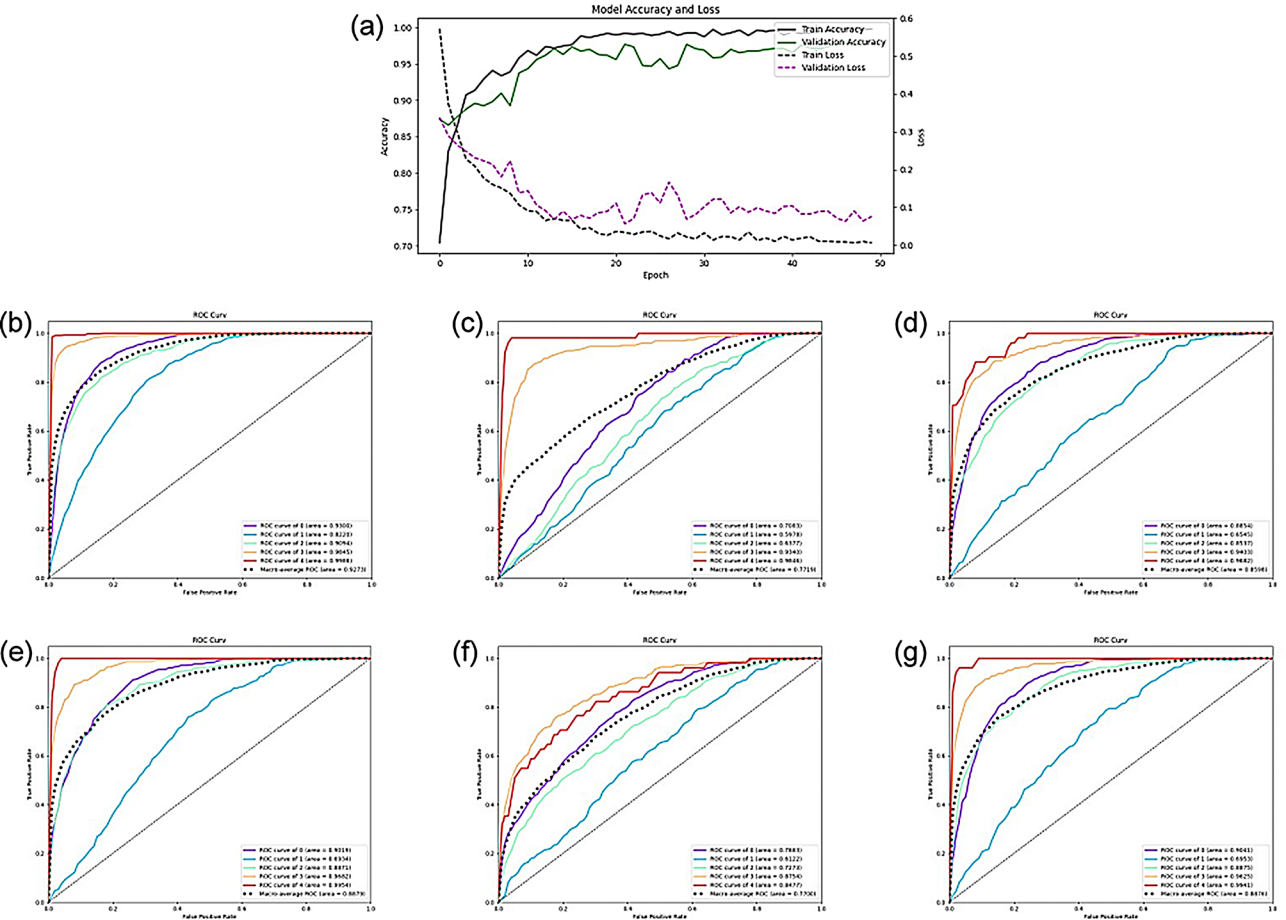
The result of DMS and baseline models

As delineated in Table 1, within the baseline models, DenseNet exhibited the most proficient performance with an average ACC of 63.52% and AUC of 88.79%. However, the acc of DenseNet is still lagged behind the DMS model by over 9%, highlighting the superior capabilities of the DMS model. The ROC curves of the baseline models are illustrated in Fig. 5(c)-(g). Notably, none of the baseline models achieved over 90% accuracy in predicting grade 3 classifications. In contrast, the DMS model demonstrated significant improvements, particularly in the accurate classification of more severe grades of Knee Osteoarthritis (OA). This accuracy renders the DMS model an instrumental tool for physicians in aiding diagnostic and treatment choices, helping patients select the appropriate treatment, reducing the burden on radiologists, and importantly, minimizing the rate of misdiagnosis to significantly reduce patient detriment.

### Visualization and clinical interpretive analysis

**Fig. 6:**
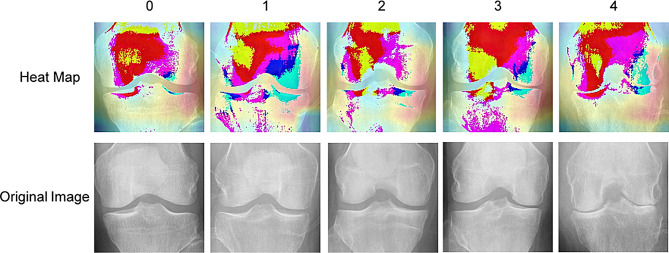
The class activation heatmap

To enhance the interpretability of our model and garner greater trust from both physicians and patients in our proposed DMS model, this study extracted the output of the final convolutional layer, creating a visualization akin to a thermal activation map. As illustrated in Fig. 6, these images vividly demonstrate how the model accurately identifies key areas for assessment in different gradations of Knee OA, subsequently making precise classifications. This process not only confirms the efficacy of the model but also provides valuable visual evidence for clinical decision-making.

## Discussion

To accurate detect Knee OA and reduce the misdiagnosis rate, facilitating doctors in diagnosis and aiding patients in choosing suitable treatment options, this study introduces a novel deep learning architecture named the DMS model. Following unseen-data validation and comparative analysis with baseline models, the DMS model demonstrates a significant performance advantage. Specifically, its average AUC value has improved by over 4%, achieving 92.73%. This accomplishment not only holds substantial clinical value but also indicates the broad applicability of the DMS model in clinical diagnosis and grading of osteoarthritis, potentially serving as a vital tool for radiologists.

The DMS model proposed in this study is realized by incorporating DenseNet’s dense connection strategy within MSC. The novelty of the DMS model lies in its improved parameter efficiency, feature reuse and gradient flow. Compared to existing methods, DMS achieves more efficient feature reuse and reduces the number of parameters through dense connections, while mitigating the gradient vanishing problem through cross-layer connections. Dense connections are employed in 1 × 1, 3 × 3, and 5 × 5 convolutions to enhance feature reuse, enabling the model to capture more complex and finer features, thereby further enhancing its recognition capabilities. Moreover, dense connections between convolutional layers contribute to constructing a deeper network structure while avoiding the issue of gradient vanishing. In the task of predicting Knee OA using knee joint X-ray images, where images of different grades are quite similar, the model needs to focus on more subtle texture features for judgment. The MSC and dense connection strategies introduced in this study effectively achieve this improvement, thus leading to exceptional performance.

Notably, based on our proposed Grad-CAM visualizations, we gain a deeper understanding of how the DMS model provides valuable auxiliary information in the grading of OA. Clinical diagnosis of OA typically focuses on specific areas of change and pathological signs. The heat maps illustrate the crucial areas the model focuses on across various grades of OA, signifying significant importance for clinical decision-making. Primarily, in OA grades 1 and 2, the model’s focal points predominantly concentrate on alterations in the joint space and regions of cartilage degeneration. This aligns with the typical manifestations observed in early-stage OA clinically. The heightened intensity in these areas indicates the model’s sensitivity to joint space narrowing and cartilage reduction, common features in early-stage OA. Subsequently, in grades 3 and 4, the model’s heat maps reveal more extensive and prominent areas of pathology. Apart from significant changes in the joint space and cartilage, the model emphasizes on osteophyte formation, synovial inflammation, and damage to periarticular soft tissues. This strongly correlates with the characteristic features seen in moderate to advanced-stage OA clinically. The heightened intensity in these areas signifies the model’s attention to bone pathology and the degree of inflammation, providing further detailed insights for diagnosis and treatment.

Furthermore, the high-temperature regions in the model’s heat maps align with the visual analysis and professional expertise of clinical practitioners. These areas of model attention often correspond to the initial focus of clinical practitioners when examining X-ray images. Such consistency demonstrates the relevance and reliability of the DMS model in identifying various grades of OA concerning clinical practice. By highlighting specific areas of change, the model offers clinicians more detailed information, aiding in a more precise determination of OA grades and the extent of pathology. This amalgamation of model outputs and clinical expertise holds promise in refining the diagnostic process of OA, thereby enhancing diagnostic accuracy and credibility, and ultimately providing patients with more personalized and precise treatment strategies.

Although our model has undergone significant structural improvements, it still confronts certain limitations. While the proposed model has enhanced the extraction of detailed features in Knee OA CT images to some extent, it struggles with capturing and focusing on some subtle textural features. This limitation results in the DMS model’s performance in predicting mild osteoarthritis (class 1) being improved compared to traditional models but still lacking. Recognizing these detailed textures remains a substantial challenge in the field, necessitating the development of new model architectures to address this issue.

Regarding the dataset, firstly, the test database used is relatively homogeneous and lacks the support of a broad standard database, limiting the model’s general applicability. Future work should focus on integrating a more diverse range of data sources to ensure wider applicability of the model. Secondly, there may be inaccuracies in the annotations of the current training dataset, which could potentially affect the model’s performance. To enhance accuracy, future efforts could involve increasing the number of training images and expanding the expert panel for more rigorous categorization. Thirdly, due to a lack of extensively manually annotated medical images by experts, we encounter challenges in assessing the realism of the computer model’s performance [33]. Lastly, our model is currently in the theoretical testing stage and requires further development to achieve full integration into hospital systems.

## Conclusion

In this study, we introduced a model named DMS, aimed at enhancing the diagnostic efficiency and reducing the misdiagnosis rate of Knee Osteoarthritis (Knee OA), thereby facilitating effective examinations. Initially, a series of feature extraction processes were applied to the raw data to minimize noise interference and enhance the expression of texture information. These processes make the model more adept at recognizing key features, thus improving its predictive performance. Subsequently, we developed the DMS model, a novel deep learning architecture that integrates Multi-Scale Convolution (MSC) and dense connections. Dense connections were not only incorporated between convolutional layers but also among convolutions of varying sizes within the same layer, to enhance the model’s ability to extract subtle features and facilitate feature sharing. Comparative analyses with unseen-data testing and baseline models demonstrated that our proposed DMS model exhibits exceptional performance, achieving an AUC of 92.37%. This achievement indicates the potential application of the DMS model in clinical settings, assisting physicians in diagnosing and grading Knee OA, selecting appropriate treatment methods, and ultimately reducing the rate of misdiagnosis, thereby alleviating the burden on patients.

## Data Availability

The data presented in this study are available in Osteoarthritis Initiative (OAI) database.
